# Rapid Detection of Fungi in Nail Specimens Using Multi‐Tube Recombinase Polymerase Amplification Assay

**DOI:** 10.1111/myc.70162

**Published:** 2026-02-14

**Authors:** Rong Wu, Meirong Li, Weian Du, Zetao Chen, Songchao Yin, Ling Hu, Liyan Xi, Jing Zhang, Huaiqiu Huang

**Affiliations:** ^1^ Department of Dermatology, The Third Affiliated Hospital Sun Yat‐Sen University Guangzhou China; ^2^ Department of Dermatology, Sun Yat‐Sen Memorial Hospital Sun Yat‐Sen University Guangzhou China; ^3^ Guangdong Provincial Key Laboratory of Malignant Tumor Epigenetics and Gene Regulation, Medical Research Center, Sun Yat‐Sen Memorial Hospital Sun Yat‐Sen University Guangzhou China

**Keywords:** Bayesian latent class analysis, dermatophytes, isothermal amplification, onychomycosis, rapid detection, recombinase polymerase amplification

## Abstract

**Background:**

Conventional diagnostic methods for onychomycosis possess disadvantages related to limited sensitivity, extended turnaround times and strong reliance on operator expertise.

**Objectives:**

To develop a molecular assay with high sensitivity and specificity for rapid detection of multiple fungal pathogens in nail specimens.

**Methods:**

A multi‐tube recombinase polymerase amplification (RPA) assay with fluorescent signal detection was developed, targeting pan‐fungal, *Trichophyton rubrum*, *T. interdigitale*, pan‐*Candida*, *Candida albicans* , pan‐*Trichosporon* and pan‐*Aspergillus*. The analytical sensitivity and specificity of the assay were evaluated using fungal strains, and its performance was assessed in 81 clinical nail specimens in parallel with calcofluor white fluorescence microscopy and fungal culture.

**Results:**

The multi‐tube RPA assay demonstrated a detection limit of 1 pg of genomic DNA for all targets, with a turnaround time of < 70 min. In the evaluation of 81 clinical nail specimens, Bayesian latent class analysis estimated sensitivities of 86.0% for multi‐tube RPA, 67.0% for fungal culture and 77.0% for microscopy, with corresponding specificities of 85.0%, 89.0% and 87.0%. The positive and negative predictive values were 92.0% and 72.0% for multi‐tube RPA, 93.0% and 55.0% for fungal culture, and 93.0% and 66.0% for microscopy.

**Conclusion:**

Compared with fungal culture and microscopy, the multi‐tube RPA assay demonstrated superior sensitivity while maintaining good specificity, facilitating the identification of fungal pathogens at the species level in nail specimens. Its operational simplicity, enhanced diagnostic performance and reduced processing times make it a promising alternative for the mycological diagnosis of onychomycosis.

## 1 Introduction

Onychomycosis is a prevalent fungal infection of the nails, affecting approximately 5.5% of the general population, with an increased incidence in individuals with diabetes or immunosuppression [1]. This condition is caused by infections from dermatophytes, non‐dermatophyte moulds and yeasts, with dermatophytes accounting for 60%–70% of these infections [2].

Onychomycosis constitutes approximately 50% of all nail disorders [3]. Trauma, psoriasis and lichen planus can also induce nail alterations similar to those observed in onychomycosis, underscoring the need for accurate and rapid diagnosis of onychomycosis [4]. Conventional diagnostic methods, such as direct microscopy, fungal culture and histopathological examination, remain prevalent in clinical practice, but each method has inherent limitations [5]. Microscopy, although rapid, is highly dependent on operator expertise and lacks species‐level resolution; culture facilitates pathogen identification, but requires prolonged incubation, often taking several days to weeks, and is susceptible to false negatives; histopathology provides structural insights, but is invasive and less effective in infections with low fungal burden [6]. Collectively, these limitations compromise diagnostic accuracy and delay the initiation of appropriate antifungal therapy, underscoring the need for improved diagnostic strategies.

Molecular diagnostic techniques, particularly those based on polymerase chain reaction (PCR), have significantly enhanced the sensitivity and specificity of fungal detection in nail specimens [7]. However, their reliance on expensive thermal cyclers, trained personnel, and extended processing times limits their feasibility in low‐resource or point‐of‐care settings. In contrast, isothermal nucleic acid amplification methods eliminate the need for thermal cycling, offering simplified workflows and rapid results that are appropriate for point‐of‐care testing [8, 9]. Among these methods, recombinase polymerase amplification (RPA) is an emerging isothermal platform that operates efficiently at a constant low temperature (typically 37°C–42°C), produces results within approximately 20 min and demonstrates strong tolerance to inhibitors commonly present in clinical samples [10]. Despite these advantages, the application of RPA for directly detecting fungi responsible for onychomycosis in nail specimens remains underexplored.

**FIGURE 1:**
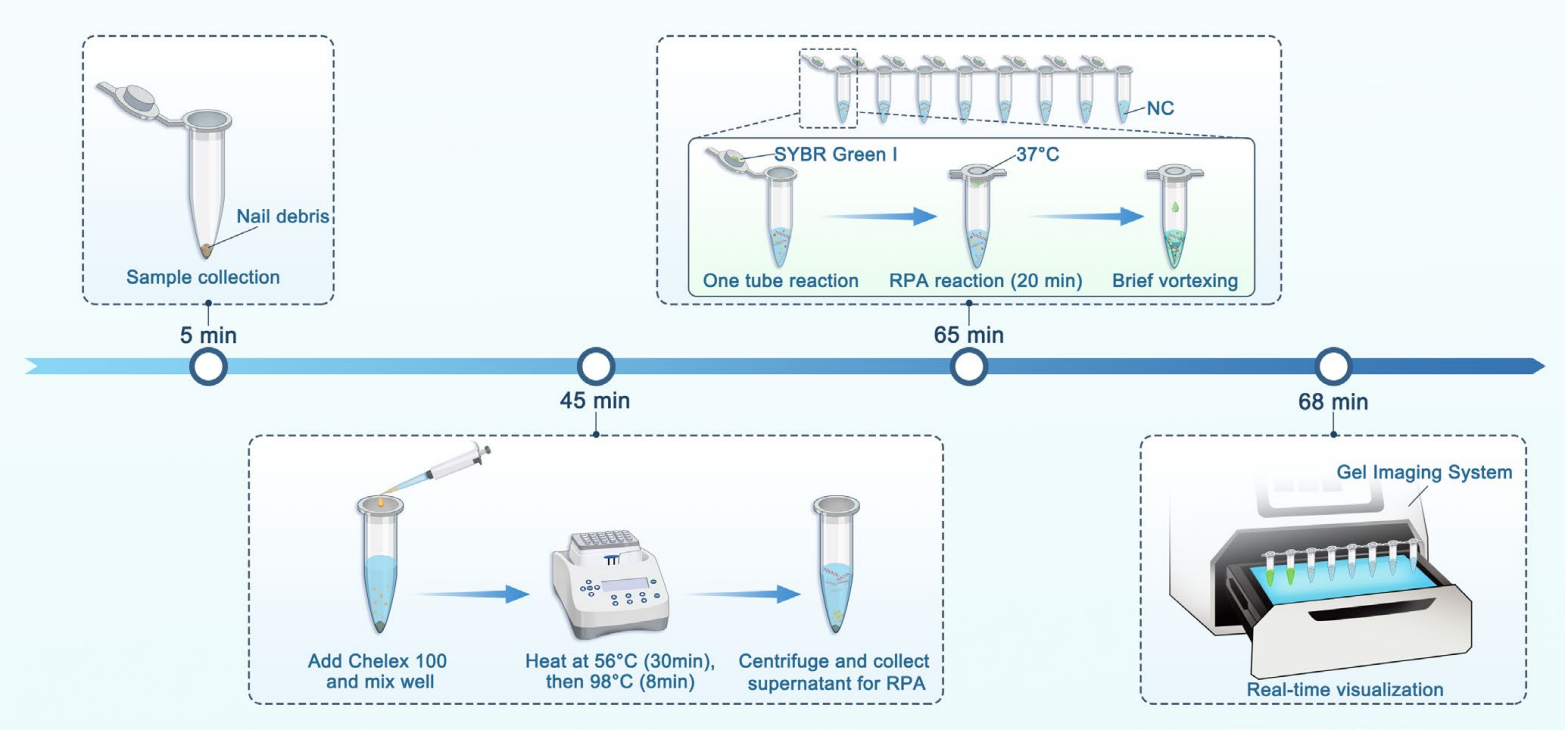
Schematic illustration of a multi‐tube recombinase polymerase amplification (RPA) assay. Clinical nail debris specimens were collected, and fungal DNA was extracted using Chelex 100. Parallel RPA reactions targeting multiple fungal pathogens were performed in separate tubes at 37°C, with SYBR Green I preloaded in the tube caps to enable closed‐tube fluorescence detection and minimise the risk of contamination. After incubation, the reaction contents were briefly mixed, and fluorescence signals were visualised under UV light using a gel imaging system. Positive reactions produced clearly distinguishable fluorescence signals, allowing rapid and intuitive pathogen identification within 70 min. NC, no‐template control.

Herein, we developed a multi‐tube RPA assay incorporating SYBR Green I for fluorescent signal detection (Figure 1). This multi‐tube RPA system enables rapid detection directly from nail specimens covering seven targets, including pan‐fungal, *Trichophyton rubrum*, *T. interdigitale*, pan‐*Candida*, *Candida albicans* , pan‐*Trichosporon* and pan‐*Aspergillus*, with results obtained within 70 min.

## 2 Materials and Methods

### 2.1 Strains and Clinical Nail Samples Collection

To evaluate the analytical sensitivity and specificity of the multi‐tube RPA assay, a total of 35 fungal strains were included (Table 1). All strains were obtained from the Department of Dermatology of The Third Affiliated Hospital of Sun Yat‐sen University. The internal transcribed spacer (ITS) region of rDNA from each strain was amplified using the universal fungal primers ITS1 (5′‐TCCGTAGGTGAACCTGCGG‐3′) and ITS4 (5′‐TCCTCCGCTTATTGATATGC‐3′). The resulting sequences were analysed using NCBI BLAST (http://www.ncbi.nlm.nih.gov) for species identification.

**TABLE 1:** Fungal strains used for analytical sensitivity and specificity evaluation of the multi‐tube recombinase polymerase amplification assay.

No.	Category	Strains
1	Dermatophytes	*T. rubrum* (ATCC 4438)
2		*T. rubrum* (SY 19017)
3		*T. violaceum* (SY 19196)
4		*T. mentagrophytes* (SY 19042)
5		*T. interdigitale* (SY 19088)
6		*T. tonsurans* (SY 19103)
7		*Microsporum canis* (SY 19014)
8		*M. ferrugineum* (SY 19010)
9		*Epidermophyton floccosum* (SY 19051)
10		*Nannizzia gypsea* (SY 19064)
11	*Candida* spp.	*C. albicans* (SC 5314)
12		*C. albicans* (ATCC 64550)
13		*C. glabrata* (SY 19060)
14		*C. parapsilosis* (ATCC 22019)
15		*C. tropicalis* (ATCC 750)
16		*C. krusei* (SY 19061)
17	*Trichosporon* spp.	*T. asahii* (SY 19070)
18		*T. asteroides* (SY 19071)
19		*T. inkin* (SY 19072)
20		*T. japonicum* (SY 19075)
21		*T. jirovecii* (SY 19078)
22	*Fonsecaea* spp.	*F. monophora* (SY 19033)
23		*F. nubica* (SY 19034)
24	*Aspergillus* spp.	*A. flavus* (SY 19080)
25		*A. fumigatus* (SY 19081)
26		*A. terreus* (SY 19082)
27		*A. sydowii* (SY 19083)
28		*A. versicolor* (SY 19084)
29	*Sporothrix* spp.	*S. globosa* (CBS 129723)
30	*Cryptococcus* spp.	*C. neoformans* (SY 19087)
31		*C. gattii* (SY 19089)
32	*Malassezia* spp.	*M. globosa* (SY 19012)
33	*Saccharomyces* spp.	*S. cerevisiae* (ATCC 9763)
34	*Staphylococcus* spp.	*S. aureus* (ATCC 25923)
35	*Escherichia* spp.	*E. coli* (ATCC 25922)

Between August 2021 and January 2022, clinical nail samples from individuals with a clinical suspicion of onychomycosis were collected at the Department of Dermatology, The Third Affiliated Hospital of Sun Yat‐sen University. Samples were included if sufficient nail material was available for all diagnostic assays. Samples were excluded if the quantity of nail debris was inadequate, if topical antifungal agents had been applied to the target nail or periungual skin within the previous 2 weeks, or systemic antifungal agents had been administered within the previous 3 months. A total of 81 nail samples met these criteria and were included in the final analysis. A portion of each sample was used for fluorescence microscopy and fungal culture, whereas the remaining material was stored at −80°C for subsequent multi‐tube RPA testing.

### 2.2 Conventional Diagnosis

Microscopic examination was conducted using calcofluor white staining under a fluorescence microscope, and samples were considered positive when fungal hyphae and/or spores were observed. Fungal cultures were cultivated on Sabouraud dextrose agar supplemented with chloramphenicol (0.05%) and incubated at 25°C for up to 4 weeks. Species identification of all clinical isolates was performed based on macroscopic and microscopic morphological characteristics and subsequently confirmed by sequencing of the fungal ribosomal DNA ITS region.

### 2.3 DNA Extraction

Fungal isolates (5–10 mg) or nail samples (1 mg) were combined with 100 μL of 5% Chelex 100 solution in a microcentrifuge tube and thoroughly vortexed. The suspension was incubated at 56°C for 30 min, vortexed for 8 s, heated at 98°C for 8 min, vortexed again for 8 s and centrifuged at 15,000 × *g* for 3 min. The supernatant was collected and used directly as the template for subsequent amplification.

### 2.4 Design of the Multi‐Tube RPA Assay

To facilitate rapid, simultaneous detection of major onychomycosis pathogens, a multi‐tube RPA assay was developed (Table 2). Tube 1 served as the pan‐fungal reaction, targeting the ITS1–5.8S–ITS2 region using a pair of pan‐fungal primers. Tubes 2 and 3 were designed to detect *T. rubrum* and *T. interdigitale*, respectively, using a universal ITS1 forward primer paired with species‐specific reverse primers targeting the 5.8S–ITS2 region. Tube 4 targeted the ITS1–5.8S–ITS2 region of five clinically relevant *Candida* species ( *C. albicans* , *C. parapsilosis* , *C. glabrata* , *C. krusei* and *C. tropicalis* ), whereas Tube 5 specifically detected *C. albicans* . Tube 6 was designed to detect common *Trichosporon* species (*T. asahii*, *T. asteroides* , *T. inkin*, *T. japonicum* and *T. jirovecii*). Tube 7 targeted *Aspergillus* species ( *A. flavus* , *A. fumigatus* , *A. terreus* , *A. sydowii* and *A. versicolor* ). Tube 8 served as the no‐template negative control. All primers were synthesised by Sangon Biotech Co. Ltd. (Shanghai, China).

**TABLE 2:** Primers used in this study.

Assay	Primer	Sequence (5′–3′)
Pan‐fungal	Forward	GGAAGTAAAAGTCGTAACAAGGTTTCTG
	Reverse	TCCTCCGCTTATTGATATGCTTAAGTTCAG
*T. rubrum*	Forward	TCGATGAAGAACGCAGCGAAATGCGATAAG
	Reverse	TGAATTGGCTGCCCATTCGCCTAGGAAG
*T. interdigitale*	Forward	GCATTTCAGCCCCTCAAGCCCAGCTTGTGTGAT
	Reverse	CTGGCCACTGCTTTTCGGGCGCGTCCCGCAC
Pan‐*Candida*	Forward	AAACTTTCAACAACGGATCTCTTGGTTCTC
	Reverse	TTCCTCCGCTTATTGATATGCTTAAGTTCAGC
*C. albicans*	Forward	GCCGCCAGAGGTCTAAACTTACAACCAATT
	Reverse	GAGGTCAAAGTTTGAAGATATACGTGGTAGAC
Pan‐*Trichosporon*	Forward	GGTGAACCTGCGGAAGGATCATTAGTGA
	Reverse	GTAATTGTCCTTGCGGACGATTAGAAGC
Pan‐*Aspergillus*	Forward	TCAACAATGGATCTCTTGGTTCCGGCATCG
	Reverse	GGGTATCCCTACCTGATCCGAGGTCAACC

### 2.5 Multi‐Tube RPA Assay

Multi‐tube RPA reactions were conducted in 50 μL volumes utilising the TwistAmp Basic Kit (TwistDX, Cambridge, UK). Each reaction mixture comprised 2.4 μL of each primer (10 μM), 29.5 μL of rehydration buffer, 11.2 μL of nuclease‐free water and 2 μL of DNA template. To initiate the reaction, 2.5 μL of MgOAc (280 mM) was added to the bottom of the tube, while 2 μL of SYBR Green I (400×) was placed on the inner surface of the tube cap. The tubes were then incubated at 37°C for 20 min, followed by brief mixing to allow incorporation of SYBR Green I into the reaction mixture. Fluorescence signals were then visualised under UV illumination and recorded using a gel imaging system. Samples exhibiting fluorescence in the pan‐fungal tube were considered positive for fungal detection. Concurrent fluorescence in the pan‐fungal tube and a corresponding genus‐ or species‐specific tube enabled further aetiological classification. Samples without detectable fluorescence in any tube were classified as negative.

### 2.6 Analytical Sensitivity and Specificity of the RPA Assay

Analytical specificity was evaluated using genomic DNA from 35 reference strains (Table 1), with all assays performed in triplicate. Analytical sensitivity was determined by testing 10‐fold serial dilutions of genomic DNA from each corresponding target strain for each primer set. Nuclease‐free water was added to Tube 8 as the no‐template negative control.

### 2.7 Statistical Analysis

To assess the diagnostic performance of the newly developed multi‐tube RPA assay in the absence of a definitive gold standard, Bayesian latent class analysis (BLCA) was applied [11, 12]. Three diagnostic modalities (microscopy, culture and multi‐tube RPA) were jointly modelled under an assumption of conditional independence. Fixed‐effects and random‐effects models accounting for conditional dependence were also explored as part of sensitivity analyses. Posterior estimates for sensitivity, specificity and disease prevalence were summarised using medians and 95% credible interval. All models were implemented in R 4.5.1 (R Foundation for Statistical Computing, Vienna, Austria), using weakly informative priors, and model convergence was assessed using Gelman–Rubin diagnostic and Raftery–Lewis criteria. Prevalence, along with the diagnostic sensitivity, specificity, negative predictive value (NPV) and positive predictive value (PPV) of the microscopy, fungal culture and multi‐tube RPA assay, were determined based on the BLCA outcomes.

## 3 Results

### 3.1 Analytical Specificity of Multi‐Tube RPA Assay Using Fungal Strains

**FIGURE 2:**
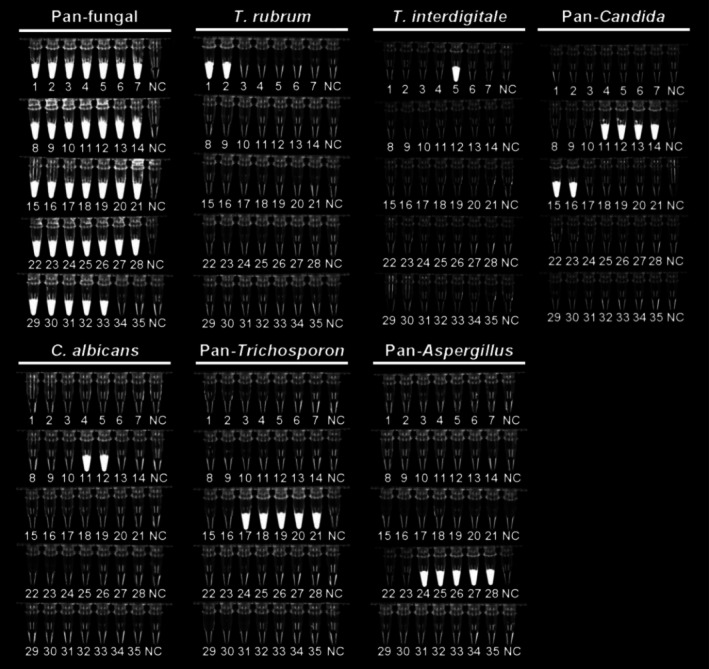
Analytical specificity of the multi‐tube recombinase polymerase amplification assay. No cross‐reactivity was observed among the 35 reference strains tested (Table 2). Fluorescence signals were observed exclusively in the corresponding target species for each primer set, whereas no amplification signals were detected in non‐target fungal strains or negative controls.

As illustrated in Figure 2, the pan‐fungal RPA primer set exhibited no cross‐reactivity with the bacterial strains tested while detecting a broad spectrum of fungal species (35 strains; Table 1). The species‐specific RPA primer sets for *T. rubrum* , *T. interdigitale* and *C. albicans* correctly identified their respective targets without cross‐detection. Similarly, the pan‐*Candida*, pan‐*Trichosporon* and pan‐*Aspergillus* RPA primer sets demonstrated high specificity across diverse fungal species and produced no detectable signal in bacterial samples.

**FIGURE 3:**
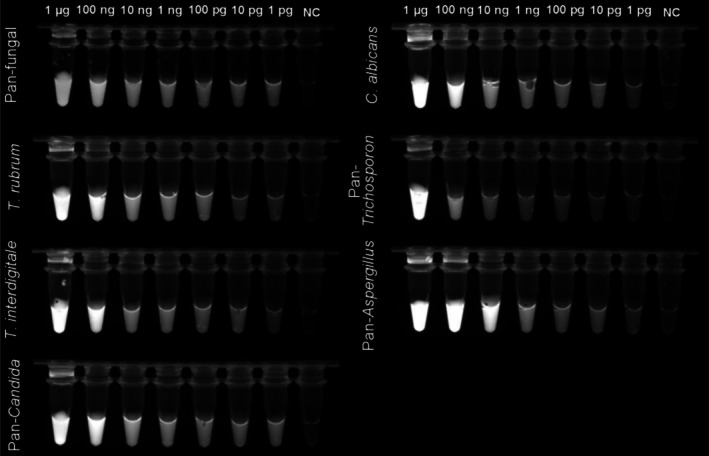
Analytical sensitivity of the multi‐tube recombinase polymerase amplification assay. Tenfold serial dilutions of genomic DNA from each corresponding target were amplified using the respective RPA assays. Fluorescence signals were recorded under UV illumination, and the lowest DNA input producing a clearly detectable signal was defined as the limit of detection. NC, no‐template control.

The analytical sensitivity of the multi‐tube RPA assay was evaluated using 10‐fold serial dilutions of genomic DNA from representative fungal pathogens. As depicted in Figure 3, all seven RPA primer sets (pan‐fungal, *T. rubrum* , *T. interdigitale*, pan‐*Candida*, *C. albicans* , pan‐*Trichosporon* and pan‐*Aspergillus*) yielded readily distinguishable fluorescence signals at DNA inputs as low as 1 pg per reaction. No fluorescence was observed in the no‐template controls. Accordingly, the limit of detection for all targets was determined to be 1 pg of genomic DNA per reaction.

### 3.2 Clinical Evaluation of Multi‐Tube RPA Assay Using DNA Extracted From Nail Specimens

A total of 81 clinical nail specimens underwent parallel testing using fluorescence microscopy, fungal culture and multi‐tube RPA (Table 3). Fluorescence microscopy yielded positive results in 75.3% (61/81) of samples, including 30 samples that were negative by culture. Fungal culture demonstrated positivity in 51.9% (42/81) of the samples, of which 34 were dermatophytes, including *T. rubrum* (*n* = 30), *T. mentagrophytes* (*n* = 3) and *T. interdigitale* (*n* = 1). The remaining eight culture‐positive samples contained non‐dermatophyte fungi, including *C. albicans* (*n* = 3, two mixed with *T. rubrum* ), *C. glabrata* (*n* = 2), *C. parapsilosis* (*n* = 1), *T. asahii* (*n* = 1), *A. sydowii* (*n* = 1), *A. versicolor* (*n* = 1) and *Fusarium solani* (*n* = 1).

**TABLE 3:** Results of microscopy, fungal culture and multi‐tube recombinase polymerase amplification in 81 nail samples.

Microscopy	Fungal culture	Multi‐tube RPA	No. of samples
Negative	Negative	Negative	6
Positive	Negative	Negative	17
Negative	Positive	Negative	0
Negative	Negative	Positive	3
Positive	Positive	Negative	0
Positive	Negative	Positive	13
Negative	Positive	Positive	11
Positive	Positive	Positive	31

Overall, 71.6% (58/81) of the samples tested positive using multi‐tube RPA assay, including 47 samples identified as dermatophytes ( *T. rubrum* , *n* = 46; *T. interdigitale*, *n* = 1). Of the 34 samples that yielded dermatophytes through culture, 55.9% (19/34) were detected by RPA and 47.1% (16/34) were deemed positive by microscopy. Conversely, among the 47 samples identified as dermatophyte‐positive by RPA, 87.2% (41/47) were corroborated by microscopy. All eight culture‐positive non‐dermatophyte samples were detected by the multi‐tube RPA assay. Among these, six samples (*F. solani*, *T. asahii*, *A. sydowii*, *A. versicolor* , *C. albicans* and *C. glabrata* ) were positive by both culture and RPA, but negative by microscopy, whereas the remaining two samples ( *C. glabrata* and *C. parapsilosis* ) were positive by all three methods. Notably, three samples that were negative by both microscopy and culture were detected as dermatophyte‐positive by RPA.

Bayesian latent class models were employed to estimate the diagnostic performance of microscopy, fungal culture and multi‐tube RPA assay in the absence of a perfect reference standard (Table 4). Estimates of sensitivity, specificity and predictive values were generally consistent across the three modelling strategies, indicating the robustness of the results. Therefore, a random‐effects model, which allows test correlations to vary across individuals, was used as the primary model for inference. BLCA estimated the prevalence in this cohort to range from 0.68 to 0.71. Microscopy demonstrated moderate sensitivity (0.75–0.81) with high specificity (0.85–0.87), whereas fungal culture exhibited lower sensitivity (0.63–0.68) but the highest specificity (0.89–0.89). In contrast, the multi‐tube RPA assay achieved the highest sensitivity across all models (0.84–0.86), with reasonably high specificity (0.82–0.85). Predictive values were calculated using the random‐effects model at an estimated prevalence of 69.0%. All three methods exhibited high PPVs (≥ 0.92). However, the RPA assay yielded the highest NPV (0.72), followed by microscopy (0.66), whereas fungal culture demonstrated the lowest NPV (0.55).

**TABLE 4:** Posterior medians and 95% credible interval for prevalence and diagnostic performance estimated using Bayesian latent class models.

Parameter	Conditional independent model	Fixed‐effects model	Random‐effects model
Prevalence	0.68 (0.54–0.79)	0.71 (0.58–0.83)	0.69 (0.56–0.82)
Microscopy			
Sensitivity	0.81 (0.69–0.90)	0.75 (0.64–0.85)	0.77 (0.65–0.87)
Specificity	0.87 (0.71–0.95)	0.85 (0.69–0.93)	0.87 (0.71–0.95)
PPV	0.93 (0.83–0.98)	0.93 (0.84–0.98)	0.93 (0.88–0.97)
NPV	0.67 (0.48–0.83)	0.60 (0.39–0.78)	0.66 (0.58–0.73)
Fungal culture			
Sensitivity	0.68 (0.54–0.80)	0.63 (0.51–0.76)	0.67 (0.53–0.79)
Specificity	0.87 (0.74–0.93)	0.87 (0.72–0.94)	0.89 (0.74–0.95)
PPV	0.94 (0.84–0.99)	0.94 (0.84–0.98)	0.93 (0.86–0.97)
NPV	0.57 (0.39–0.74)	0.50 (0.32–0.68)	0.55 (0.48–0.62)
Multi‐tube RPA			
Sensitivity	0.84 (0.72–0.92)	0.86 (0.74–0.94)	0.86 (0.75–0.94)
Specificity	0.85 (0.69–0.94)	0.82 (0.66–0.92)	0.85 (0.69–0.95)
PPV	0.92 (0.82–0.97)	0.92 (0.83–0.97)	0.92 (0.88–0.96)
NPV	0.70 (0.50–0.86)	0.67 (0.45–0.84)	0.72 (0.64–0.79)

Abbreviations: NPV, negative predictive value; PPV, positive predictive value.

## 4 Discussion

Accurate mycological testing is crucial for the diagnosis and effective management of onychomycosis. An optimal mycological assay should rapidly and accurately identify pathogens while maintaining high sensitivity and specificity [13]. In this study, we developed a multi‐tube RPA assay incorporating primer sets targeting pan‐fungal, *T. rubrum* , *T. interdigitale*, pan‐*Candida*, *C. albicans* , pan‐*Trichosporon* and pan‐*Aspergillus*. By integrating Chelex 100 DNA extraction, low‐temperature isothermal amplification and fluorescence detection, this assay facilitates the accurate and rapid identification of nail infection pathogens.

Molecular diagnostic methods are extensively employed for fungal identification in clinical practice and generally outperform conventional methods. The 2023 S1 guideline recommends PCR as a key adjunct to traditional mycological diagnostic methods [4, 14]. Several PCR‐based approaches have been developed for the diagnosis of onychomycosis, including multiplex PCR [15], reverse blot hybridisation PCR assays [16], loop‐mediated isothermal amplification [17] and real‐time PCR platforms utilising molecular beacons or TaqMan probes [18, 19]. While commercial real‐time PCR assays have reduced the turnaround time from sample request to result reporting from 19 days to approximately 16 h [20], the multi‐tube RPA assay enables a further reduction to approximately 70 min. Real‐time PCR has been reported to achieve detection limits ranging from approximately 100 fg to 5 pg of genomic DNA per reaction [21, 22], whereas loop‐mediated isothermal amplification assays exhibit detection limits on the order of 10 ng per reaction [17]. In comparison, the multi‐tube RPA assay achieved a detection limit of 1 pg per reaction. In clinical sample testing, the multi‐tube RPA assay achieved an 80.2% (65/81) concordance with fungal culture, comparable to the performance of the dermatophyte multiplex qPCR assay [23]. Both assays demonstrated higher sensitivity than conventional culture methods.

In our study, fungal pathogens in culture‐confirmed onychomycosis were predominantly dermatophytes (34/42), with *T. rubrum* being the most common (30/42), followed by *T. mentagrophytes* (3/42) and *T. interdigitale* (1/42). Notably, two cases yielded concurrent isolation of *T. rubrum* and *C. albicans* . Additionally, a small number of cases were caused by non‐dermatophyte moulds (3/42) and yeasts (5/42). These findings are generally consistent with epidemiological data from mainland China, where dermatophytes predominate (60.59%), while yeasts (30.09%), moulds (7.91%) and mixed infections (1.41%) also contribute substantially, particularly in the warm and humid southern regions [2, 24]. Hence, the newly developed multi‐tube RPA assay enables species‐level identification and is potentially applicable to the majority of onychomycosis cases.

Furthermore, the multi‐tube RPA assay showed complete concordance with fungal culture, detecting all nail specimens that were positive by culture (100%, 42/42). Importantly, it also identified additional dermatophyte infections (16/16) that were culture‐negative, indicating its enhanced sensitivity in detecting dermatophytes responsible for onychomycosis. In comparison, the positive concordance rate between microscopy and the multi‐tube RPA assay was 72.1% (44/61), reflecting the relatively higher positivity rate of microscopic examination. Among these nail specimens, 17 samples identified as positive by microscopy but negative by the multi‐tube RPA assay likely contained structures mimicking fungal hyphae [1], degraded fungal DNA or fungal loads below the detection limit of the multi‐tube RPA assay. Additionally, microscopy cannot identify fungal genera or species. For instance, two samples that were positive by microscopy were identified as *Aspergillus* spp. by both culture and the multi‐tube RPA assay. The ability of the multi‐tube RPA assay to achieve species‐level identification is clinically relevant, as it enables differentiation between dermatophytes and non‐dermatophyte fungi, thereby guiding appropriate antifungal treatment selection [25, 26].

Given the inherent limitations of conventional culture and microscopy [13], these methods were not adopted as the sole reference standard for evaluating the diagnostic performance of the multi‐tube RPA assay. Therefore, Bayesian latent class analysis was applied to estimate diagnostic performance without assuming perfect test accuracy. The multi‐tube RPA assay demonstrated a sensitivity of 86.0% and a specificity of 85.0% for detecting fungal pathogens in clinical nail specimens. In comparison, fungal culture exhibited a sensitivity of 67.0% and a specificity of 89.0%, whereas microscopy achieved a sensitivity of 77.0% and a specificity of 87.0%. These findings are similar to previous reports on recombinase polymerase amplification‐lateral flow dipstick assays for dermatophyte detection [27]. Overall, the multi‐tube RPA assay consistently demonstrated the highest sensitivity while maintaining moderately high specificity.

Collectively, these results indicate that the multi‐tube RPA assay provides a rapid, sensitive and broadly applicable alternative to conventional mycological methods. Its ability to detect dermatophytes, fungi other than dermatophytes and mixed infections supports its potential utility for accurate diagnosis and clinical management of onychomycosis.

## Author Contributions

**Rong Wu:** conceptualisation, methodology, software, data curation, formal analysis, writing – original draft, investigation, visualisation. **Meirong Li:** data curation, investigation, methodology, validation. **Weian Du:** conceptualisation, methodology, funding acquisition. **Zetao Chen:** methodology, software, data curation, validation. **Songchao Yin:** data curation, investigation, resources. **Ling Hu:** methodology, validation. **Liyan Xi:** resources, supervision. **Jing Zhang:** project administration, writing – review and editing, supervision, resources. **Huaiqiu Huang:** supervision, funding acquisition, writing – review and editing, formal analysis.

## Ethics Statement

Nail samples for this study were leftovers of routinely processed samples and collected with approval of the ethics committee of the Third Affiliated Hospital of Sun Yat‐sen University (Ethic Number: [2021]02‐319‐01).

## Conflicts of Interest

The authors declare no conflicts of interest.

## Data Availability

The data supporting the findings of this study are available from the corresponding author upon reasonable request.
